# A quantitative perspective to the study of brain arterial remodeling of donors with and without HIV in the Brain Arterial Remodeling Study (BARS)

**DOI:** 10.3389/fphys.2014.00056

**Published:** 2014-02-19

**Authors:** Jose Gutierrez, Gorazd Rosoklija, Jacinta Murray, Christina Chon, Mitchell S. V. Elkind, James Goldman, Lawrence S. Honig, Andrew J. Dwork, Susan Morgello, Randolph S. Marshall

**Affiliations:** ^1^Department of Neurology, College of Physicians and Surgeons, Columbia University Medical CenterNew York, NY, USA; ^2^Department of Psychiatry, College of Physicians and Surgeons, Columbia University Medical CenterNew York, NY, USA; ^3^Macedonia Academy of Science and ArtsSkopje, Macedonia; ^4^Department of Neurology, Icahn School of Medicine at Mount SinaiNew York, NY, USA; ^5^Department of Epidemiology, Mailman School of Public Health, Columbia University Medical CenterNew York, NY, USA; ^6^Department of Pathology and Cell Biology, College of Physicians and Surgeons, Columbia University Medical CenterNew York, NY, USA

**Keywords:** brain arteries, arterial remodeling, media thickness, stenosis, HIV, cardiovascular disease

## Abstract

Mechanisms underlying brain arterial remodeling are uncertain. We tested the hypothesis that arterial size and location are important determinants of arterial characteristics. We collected large and penetrating brain arteries from cadavers with and without HIV. Morphometric characterization was obtained from digital images using color-based thresholding. The association of arterial size and location with lumen diameter, media and adventitia area, media proportion, a wall thickness, wall-to-lumen ratio and stenosis was obtained with multilevel mixed models and a *P* value ≤ 0.05 was considered significant. We included 336 brains, in which 2279 large arteries and 1488 penetrating arteries were identified. We found that arterial size was significantly associated with all arterial characteristics studied of large and penetrating arteries with exception of arterial stenosis in large arteries. After adjusting for size, an independent association was found between lumen diameters, media and adventitia thickness with artery locations. Arterial stenosis was also associated with artery location in both large and penetrating arteries. In summary, significant effects of size and/or location were found in arterial characteristics typically used to define arterial remodeling. Brain arterial remodeling characteristics differ across arterial sizes and location, and these differences should be controlled for in future studies of brain arterial remodeling.

## Introduction

Arterial remodeling is the process in which arteries undergo structural and functional changes upon exposure to biological stimuli including mechanical and chemical factors. Arterial remodeling represents an adaptive response that leads to different phenotypes, including thickening or thinning of the arterial wall, lumen dilatation or narrowing of the lumen, or combinations of these (Gibbons and Dzau, 1994). This adaptive response can sometimes turn pathological and cause disease. For example, atherosclerosis, a form of inward remodeling, is associated with myocardial infarction (MI) and stroke, two of the leading causes of mortality and disability in the US (Mosca et al., 2013). Progressive dilatation of the aorta can lead to aortic aneurysm, a form of outward remodeling, that can rupture and cause death (Mcmillan et al., 1997). Although less well studied, there is evidence suggesting that progressive dilatation of brain arteries, i.e., dolichoectasia (DE), is associated both with compressive effects on adjacent brain tissue and greater risk of cerebrovascular events (Passero and Rossi, 2008; Gutierrez et al., 2011a,b). It is thought that vascular risk factors play a role in the development of DE, but it can also occur in their absence (Gutierrez et al., 2011a,b). Understanding the mechanisms underlying arterial remodeling offers an opportunity to understand the physiopathology of arterial disease and to identify pathways that can lead to new, targeted therapeutic interventions.

Infection with HIV has also been associated with DE (Kossorotoff et al., 2006; Eugenin et al., 2008; Goldstein et al., 2010; Gutierrez and Ortiz, 2011). The mechanisms underlying this association are not fully understood. There is evidence, however, that the in the era of effective anti-retroviral (ARV) therapy, the aging population with HIV infection has an increasing prevalence of vascular events and dementia as a major cause of morbidity and mortality (Hassler, 1962; Connor et al., 2000; Triant et al., 2007; Ovbiagele and Nath, 2011). Multiple mechanisms have been invoked to try to explain this phenomenon. For example, some investigators have found an increased prevalence of traditional vascular risk factors in the HIV population compared to non-HIV controls (Guaraldi et al., 2011; Gutierrez et al., 2013a,b). The use of some ARV is associated with dyslipidemia and with the subsequent development of MI (Friis-Moller et al., 2003; Worm et al., 2010). Direct viral infection of the arterial wall and myocardium has been invoked as the cause of HIV vasculopathy and cardiomyopathy in adults and children (Herskowitz et al., 1994; Eugenin et al., 2008). We and others have demonstrated pathological evidence of brain arterial remodeling associated with HIV, but the small samples in these studies preclude a firm conclusion about the specificity for this change to HIV or other HIV-related co-morbidities (Eugenin et al., 2008; Gutierrez et al., 2012a,b). However, prior studies have not systematically controlled for expected differences in arterial characteristics according to their size, which presumably might lead to estimates errors.

We assembled a collection of cadaveric large and penetrating (i.e., small) brain arteries, the Brain Arterial Remodeling Study (BARS), with the overall purpose of studying the mechanisms underlying brain arterial remodeling related to aging and to vascular risk factors, with particular focus on the ways in which HIV infection alters this remodeling. The goal of this initial analysis is to test the hypothesis that arterial size (as determined by interadventitial diameter) and location (e.g., internal carotid vs. middle or anterior cerebral artery) are significant determinants of lumen diameter, media thickness, media proportion, adventitia thickness, wall thickness, wall-to-lumen ratio and degree of stenosis that may need to be considered in future studies of brain arterial remodeling.

## Materials and methods

**Figure 1:**
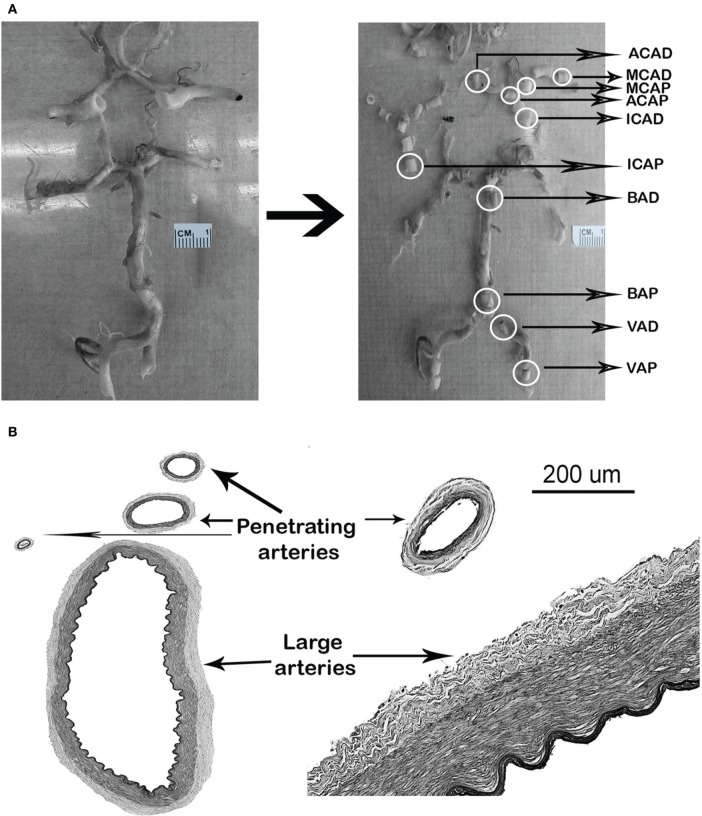
**Examples of arterial identification. (A)** Circle of Willis, identification, and section of arterial segments. **(B)** Identification of penetrating arteries neighboring the parent large artery. D, distal; P, proximal.

Arteries were obtained from 336 brains, from which 2279 large brain arteries and 1488 penetrating arteries were collected (Figure 1). The brains were obtained from four different sources of tissue collection, including the Manhattan HIV Brain Bank (MHBB) at the Mount Sinai School of Medicine (*N* = 189), the Macedonian/New York Psychiatric Institute Brain collection (*N* = 104), the New York Brain Bank/Alzheimer's Disease Research Center at Columbia University (*N* = 25), and the Brain Endowment Bank at University of Miami (*N* = 18). The descriptions of each tissue collection, the population from which the brains were obtained, and the variable definitions are listed in Table 1. Two clinical groups of interest were identified as cases: individuals with HIV (*N* = 138) and individuals with pathologically-confirmed Alzheimer disease (AD) as a model of pathological aging (*N* = 25). HIV(−) and AD(−) controls were matched 1:1 by age (±5 years) and sex, where possible, to the cases. All brain sources had approval by the IRB at their respective institutions.

**Table 1:** **Characteristics of the tissue repositories from which the brain arteries were obtained**.

	**Manhattan HIV Brain Bank (MHBB)**	**Macedonian/NYSPI Brain Collection**	**New York Brain Bank (NYBB)**	**Brain Endowment Bank (BEB)**
Institution	Mount Sinai School of Medicine	New York Psychiatric Institute	Columbia University Medical Center	University of Miami
Type of study	Cohort, living subjects followed prospectively until they die	Cross-sectional, retrospective data collection	Cross-sectional, retrospective data collection	Cross-sectional, retrospective data collection
Population studied	Individuals with HIV and HIV negative controls	Individuals with psychiatric diseases and negative controls	Individuals with neurodegenerative disease including AD	Individuals with neurological and psychiatric diseases and negative controls
Number of donors for this study	*N* = 189 (HIV+ = 133)	*N* = 104 (AD+ = 1, All HIV−)	*N* = 25 (All HIV−, AD+)	*N* = 18 (HIV+ = 5, all AD−)
Origin of the studied sample	Predominantly from inner city neighborhoods in Manhattan (East and Central Harlem), Bronx, and Brooklyn, NY	78% obtained from Macedonia, 22% obtained from a hospital-based autopsy service in NY	Metropolotan area of New York, NY	Metropolitan area, Miami, FL
Vascular risk factor definition	Chart review, self-report during professional interview, or inferred based on medication list	Chart review of subjects who die institutionalized, and/or psychological autopsy based on family interviews	Chart review, self-report by interview of prospective donors	Chart review, self-report by interview of prospective donors
Laboratory results	Obtained at scheduled visits during follow up	Retrieved from chart reviews when available	Retrieved from chart reviews when available	Retrieved from chart reviews when available
Website link	MHBB	M/NYPSI Brain Collection	NYBB	BEB
Age (mean ± *SD*)	50.0 ± 10.6	50.3 ± 11.9	81.9 ± 9.8	55.7 ± 23.1
Male sex (%)	70	71	44	56
Ethnicity (%)				
NH-whites	24	88	88	67
NH-Blacks	41	7	0	33
Hispanics	35	5	12	0

Abbreviations: AD, Alzheimer Dementia; NH, non-Hispanic; SD, standard deviation; NYSPI, New York Psychiatric Brain Institute.

The components of the circle of Willis were identified in brains in 10% buffered formalin and dissected from the brain parenchyma for better visualization and handling. Five-millimeter transverse arterial blocks were obtained from the proximal (i.e., closer to the heart) and distal (just proximal to a bifurcation) locations of the supraclinoid internal carotid artery (ICA), the first segments of the middle, anterior, and posterior cerebral arteries, the basilar artery (BA) and the intracranial portion of the vertebral arteries (VA) according to availability from both hemispheres. An additional 5-mm arterial segment was obtained from the proximal location of the second segment of the middle cerebral artery (MCA) after its primary bifurcation (Figure 1). Arteries were embedded in paraffin and 6-μm thick cuts were obtained for H&E, elastic Van Gieson (EVG), trichrome and Congo red staining. Digital images were obtained from stained arteries using Olympus Soft Imaging Solutions software and a microscope with constant illumination, with 10× magnification and scale = 0.643 μm/pixel. Penetrating arteries were identified as smaller arteries seen in the periphery of a large artery (Figure 1).

The methods used to obtain morphometric arterial characteristics have been previously described with good-to-excellent reliability for quantitative measurements (Gutierrez et al., 2012a). Color-based thresholding was applied to EVG digital files using ImageJ software (WS Rasband, ImageJ, U.S. National Institutes of Health, Bethesda, Maryland, USA, imagej.nih. gov/ij/, 1997–2011) to quantify the areas of the lumen and arterial layers. A shrinkage-correction factor of 1.25 for areas and 1.16 for perimeter was applied to all measures obtained (Glagov et al., 1987; Stary et al., 1992). Additional arterial characteristics were obtained using standard mathematical formulas (Supplementary file). Arterial stenosis was determined using the method described by Glagov et al. (1987).

### Statistical analysis

Seven characteristics were used as dependent variables for the analysis of arterial anatomy and pathology: lumen diameter, media thickness, proportion of the wall occupied by the media (i.e., media proportion), adventitia thickness, wall thickness (i.e., intima, media and adventitia), wall-to-lumen ratio and degree of stenosis. Because large differences in size among large and penetrating arteries, stratified analysis was carried out by artery type (i.e., large vs. penetrating arteries). The assumptions of normality, linearity and homoscedasticity were assessed with histograms, skewness, kurtosis (<1.0 were considered compatible with normal distribution), and Q–Q plot. When doubt remained, we also used Kolmogorov–Smirnov test for further assessment. Data transformation was used to achieve normality or near-normality as recommended elsewhere (Nishioka et al., 1996; Tabachnick and Fidell, 2007). In light of the co-dependence among arteries obtained from the same individual and the variable number of arteries per case, we used multilevel mixed models to evaluate for a statistically significant association between the dependent variables with arterial size. If the association was found significant, then adjustment for arterial size was carried out by dividing the dependent variables by interadventitial diameter. A new model was run using artery location as a predictor of the size-adjusted estimate to see whether a lasting effect can be ascertained to arterial location independent of size. Type III effects were used to obtained beta coefficients and their standard errors. The statistical software used for the analysis was SAS software, version 9.3 (SAS Institute Inc., Cary, NC).

## Results

### Sample characteristics

The sample mean age was 52.8 ± 14.5 *SD* years (median 50, IQR 16, range 21–102), 32% were women, 22% Hispanic, 28% Black, and 50% non-Hispanic white. HIV was present in 41% (*N* = 138) and 8% (*n* = 25) had AD.

### Brain arterial anatomy

The number of large artery segments available from each case varied depending on the methods in each independent study. The number of arterial segments ranged from 1 to 18, with a mean of 7 segments per case; 90% of the cases had 4 or more segments. In 38% of the cases, homologous arteries from both hemispheres were included.

**Figure 2:**
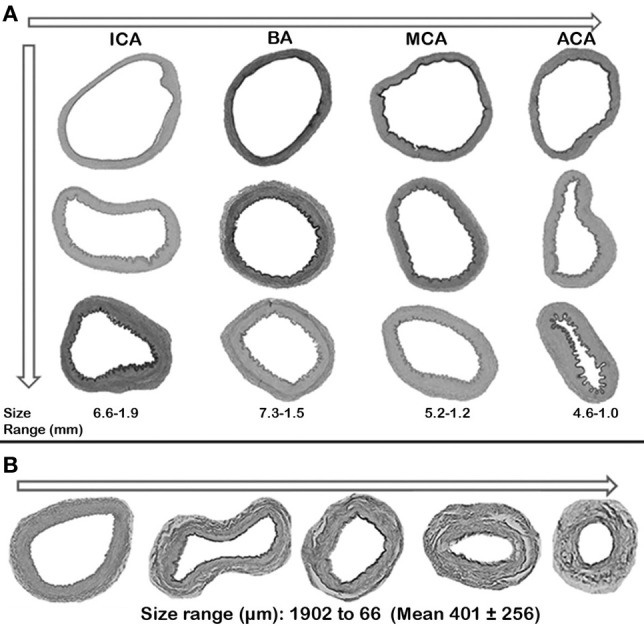
**Relationship between arterial size and wall-to-lumen ratio. (A)** Large arteries, largest to smallest. **(B)** Penetrating arteries, largest to smallest.

The interadventitial diameters of large arteries ranged from 7.8 to 1.0 mm (Mean 3.0 ± 0.9 mm). As the large arteries enter the skull and give off branches, they decrease in size. Comparing proximal vs. more distal segments of the same large artery, not only did the large arteries taper in interadventitial diameter, but the lumen and all the components of the arterial wall also became smaller. The exception to this rule appears to be the posterior cerebral artery (PCA), in which the wall is thicker distally compared to the more proximal segment (Table 2). The percentage of lumen stenosis appears greater in larger arteries, although the difference is less marked than other arterial characteristics. The relative thickness of the arterial wall compared to the lumen diameter (wall-to-lumen ratio) increases as the artery becomes more distal in most large arteries, with the exception of the ICA where the wall becomes relatively thinner compared to the lumen as it enters the brain (Figure 2A).

**Table 2:** **Arterial characteristics by arterial segment**.

**Arterial segments**	Interadventitial diameter (mm ± ***SD***)^*^	Lumen diameter (mm ± ***SD***)^*^	Media thickness (μm ± ***SD***)^*^	**Media proportion of the wall (%)**	Adventitia thickness (μm ± ***SD***)^*^	Wall thickness (μm ± ***SD***)^*^	Luminal stenosis(% ± ***SD***)^*^	Wall-to- lumen ratio (± ***SD***)^*^
Proximal ICA (supraclinoid) (*N* = 70)	3.9±0.9	2.9±0.8	200.8±68.9	40.2±9.7	159.8±60.2	529.8±232.1	19.9±14.9	6.2±2.5
Distal ICA (supraclinoid) (*N* = 238)	3.8±0.8	2.9±0.7	189.8±52.8	43.4±8.9	140.6±36.5	451.5±141.2	15.1±10.4	7.0±2.6
Proximal M1 MCA (*N* = 291)	3.1±0.7	2.3±0.6	149.5±42.7	41.1±9.1	134.4±33.7	383.6±131.8	15.4±10.8	6.6±2.2
Distal M1 MCA (*N* = 296)	2.9±0.6	2.2±0.5	141.2±37.1	41.2±8.2	124.2±32.6	357.4±131.8	14.5±9.5	6.5±2.0
Proximal M2 MCA (*N* = 264)	2.4±0.6	1.8±0.5	125.6±36.2	41.5±8.1	116.8±31.9	311.9±102.1	14.5±10.3	5.9±1.8
Proximal A1 ACA (*N* = 252)	2.4±0.6	1.7±0.5	133.4±43.9	42.9±8.4	112.5±30.4	318.7±106.2	15.8±11.8	5.8±2.1
Distal A1 ACA (*N* = 152)	2.3±0.5	1.7±0.4	124.5±47.2	43.0±8.8	106.6±26.0	296.4±97.9	13.1±7.7	6.2±2.1
Proximal V4 VA (*N* = 148)	3.2±1.2	2.3±0.9	152.0±50.9	38.4±9.9	160.2±83.0	431.3±212.9	17.8±13.1	6.1±2.5
Distal V4 VA (*N* = 220)	2.9±0.9	2.2±0.8	148.3±49.1	42.0±9.6	113.2±37.7	368.7±136.2	17.8±12.4	6.3±2.2
Proximal BA (*N* = 167)	3.7±1.0	2.9±0.8	165.5±51.9	43.7±11.0	117.0±32.7	407.0±175.8	14.7±12.3	7.8±2.9
Distal BA (*N* = 122)	3.4±0.9	2.6±0.6	165.5±52.6	45.9±9.1	109.0±32.7	377.9±145.6	13.8±10.7	7.6±2.7
Proximal P1 PCA (*N* = 43)	2.5±0.6	1.8±0.6	160.9±49.4	47.3±10.4	94.5±20.6	353.9±102.9	20.4±15.3	5.5±2.3
Distal P1 PCA (*N* = 16)	2.6±0.4	1.7±0.4	136.0±39.3	36.0±16.4	100.0±27.3	442.3±184.9	34.6±23.7	4.6±2.5

Abbreviations: mm, millimeters; um, micrometers; ICA, internal carotid artery; MCA, middle cerebral artery; ACA, anterior cerebral artery; BA, basilar artery; VA, vertebral artery; PCA, posterior cerebral artery; N, number.

* ANOVA test resulted in P ≤ 0.001 for all comparisons.

The interadventitial diameters of the penetrating arteries ranged from 66 to 1902 μm (Mean 401 ± 256 μm). The size of the penetrating arteries was directly related to the size of the parent vessel in the anterior circulation, but it was inversely related in the vertebrobasilar system. Specifically, the penetrating arteries from the ICA were larger than those of the MCA, which were larger than those of the anterior cerebral artery (ACA). The penetrating arteries from the VA, however, were larger than those of the BA despite the BA having a larger diameter than the VAs. The penetrating arteries arising from distal segments of large arteries were larger than those of the proximal segments of the same artery, with the exception of the ACA and ICA where proximal segments were actually larger (Table 3). The wall-to-lumen ratio varies by penetrating artery size. In larger penetrating arteries, the lumen is proportionally wider than in smaller penetrating arteries where the wall occupies a greater proportion of the total arterial area (Figure 2B). The absolute thickness of the media decreases as the penetrating artery becomes smaller distally but the proportion of wall thickness occupied by the media remains more stable at about a third of the total wall thickness.

**Table 3:** **Penetrating arteries characteristics by parent artery segment**.

**Penetrating arteries arising from:**	Interadventitial diameter (μm ± ***SD***)^*^	Lumen diameter (μm ± ***SD***)^*^	Media thickness (μm ± ***SD***)^*^	**Media proportion of the wall (%)**	Adventitia thickness (μm ± ***SD***)^*^	Wall thickness(μm ± ***SD***)^*^	Luminal stenosis (% ± ***SD***)^*^	Wall-to- lumen ratio (± ***SD***)^*^
Proximal ICA (supraclinoid) (*N* = 36)	365.9±265.0	238.6±205.6	19.3±16.3	29.9±10.5	37.7±22.7	59.9±12.1	13.7±5.5	3.7±1.7
Distal ICA (supraclinoid) (*N* = 142)	457.7±302.3	319.4±253.6	20.9±13.6	30.3±9.66	41.2±22.1	55.1±12.7	11.7±5.7	4.4±2.1
Proximal M1 MCA (*N* = 239)	386.3±254.4	264.5±201.7	18.4±13.4	29.8±9.03	35.8±22.2	55.7±12.5	12.4±5.9	4.4±2.3
Distal M1 MCA (*N* = 213)	431.7±281.5	299.5±229.2	21.4±14.7	31.7±9.40	37.2±18.9	55.5±11.6	12.1±4.7	4.3±2.0
Proximal M2 MCA (*N* = 126)	298.3±171.2	202.4±137.6	14.8±9.99	31.3±9.83	28.2±17.1	55.4±13.6	11.9±5.9	4.5±2.4
Proximal A1 ACA (*N* = 242)	323.1±204.0	213.5±158.5	16.8±11.2	31±9.22	32.3±19.9	58.4±11.1	13.1±5.3	3.9±2.1
Distal A1 ACA (*N* = 104)	301.7±174.3	205.1±130.2	15.9±11.0	33.5±9.82	27.1±18.3	54.4±13.2	12.3±6.7	4.6±2.3
Proximal V4 VA (*N* = 27)	452.6±158.8	321.6±132.2	21.4±9.95	31.6±8.61	35.0±10.6	50.3±10.8	11.4±5.9	5.1±1.9
Distal V4 VA (*N* = 83)	516.3±306.6	352±234.0	25.1±16.3	33.1±10.6	47.4±39.6	54.6±12.2	13.3±7.3	4.6±2.3
Proximal BA (*N* = 115)	455.8±206.9	309.6±172.9	23.4±12.7	32.3±10.4	41.0±20.8	55.3±12.8	13.3±7.1	4.4±2.0
Distal BA (*N* = 113)	537.9±282.2	387.4±248.2	26.6±12.7	36.2±10.6	39.7±22.9	50.9±12.7	11.4±5.5	5.2±2.6
Proximal P1 PCA (*N* = 35)	396.8±207.9	269±162.6	23.3±13.4	35.4±9.10	32.4±12.7	56.1±10.1	13.6±5.3	4.1±1.6
Distal P1 PCA (*N* = 13)	430.5±236.3	301.2±171.3	24.3±15.1	37±7.84	32.3±19.8	51.7±7.84	12.1±4.1	4.7±1.3

Abbreviations: mm, millimeters; um, micrometers; ICA, internal carotid artery; MCA, middle cerebral artery; ACA, anterior cerebral artery; BA, basilar artery; VA, vertebral artery; PCA, posterior cerebral artery; N, number.

* ANOVA test resulted in P ≤ 0.001 for all comparisons except for stenosis where P > 0.05.

### Relationship of artery location and size to arterial cross-section measures

We evaluated the associations between arterial size (as determined by interadventitial diameter) with each of the elements evaluated in Tables 2, 3. All the studied characteristics were significantly associated with arterial size, with the notable exception of the degree of stenosis. The estimates were consistent among the different clinical groups (Table 4). We observed that the effects of arterial size were very similar for the lumen, wall thickness and media thickness of large and small arteries. The beta coefficients for adventitia thickness, wall-to-lumen ratio and media proportion were greater for small arteries than for large arteries. Larger arteries did not have greater degree of stenosis, but a relationship between arterial size and stenosis was noted for penetrating arteries.

**Table 4:**
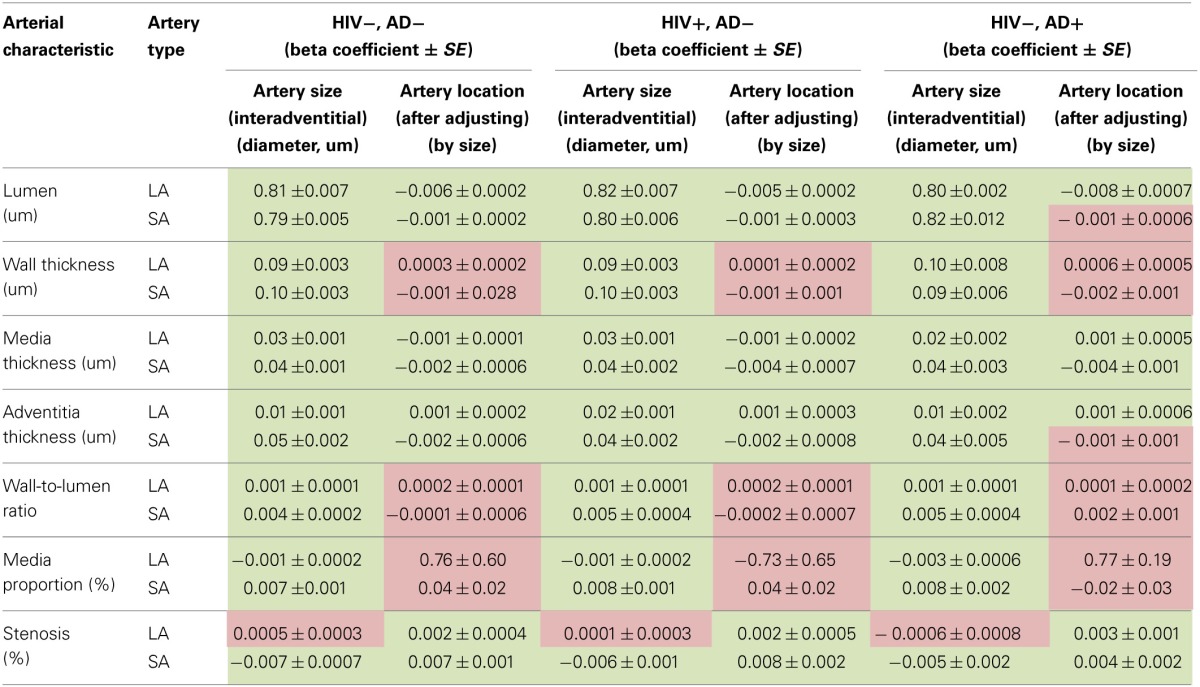
**Beta coefficients for arterial size in association with arterial characteristics by clinical group**.

Statistical significance:

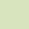
 *P-value ≤ 0.05.*

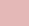
 *P-value > 0.05.*

Abbreviations: AD, Alzheimer Dementia; SE, standard error; LA, large artery; SA, small artery; um, micrometers.

Adjusting for size demonstrated an independent association between artery location with lumen diameter, media thickness and adventitia thickness in both, small and large arteries. For wall thickness, wall-to-lumen ratio and media proportion, controlling for size sufficed to explain differences among arteries with no independent effect attributed to artery location. Controlling for size disclosed an independent effect of artery location with stenosis for large arteries. For small arteries, stenosis was negatively associated with arterial size. After adjusting for size, an independent association was found between stenosis and arterial location.

## Discussion

The BARS represents a collection of large and penetrating arteries assembled to study brain arterial remodeling and its association with cerebrovascular events and dementia. Issues pertaining to the brain arterial anatomy are of great importance and should be considered when studying brain arterial remodeling, independent of the studied population. We have demonstrated that the assumptions of arterial characteristics vary significantly by size and location of the arteries studied. Because each of our cases is represented by a different number and arterial locations, we will have to use size-adjusted arterial characteristics for all aspects of remodeling presenting in this analysis and also adjust by artery location when studying the lumen, the media and adventitia thicknesses and stenosis so that estimates we obtain are reflective of the factors used to model the outcomes and not a result of the random selection of arteries included per case.

We found that the effects of size on different arterial characteristics were relatively fixed across clinical groups, which suggest that by principle, arterial size accounts for a proportion of the variance across individuals regardless of the clinical characteristics studied in this report. Whether this can be extended to other groups remains uncertain. These findings strengthen our argument that adjusting for size is of paramount importance when arteries obtained from different locations and of different sizes are analyzed. This is applicable to small and large arteries. We found that the beta estimates for lumen, wall and media absolute thicknesses were almost identical between large and small arteries. This might suggest that for any given size, there is a fixed demand in wall tensile strength as given by the wall and media thicknesses (Gasser et al., 2006). Based on the different beta coefficients of small arteries compared to large arteries, it can be inferred that the adventitia is relatively thicker, that the wall is proportionally thicker than the lumen and that the media is relatively thinner in the smallest arteries.

Arterial stenosis of large arteries was not significantly associated with arterial size *per-se*, but it was with artery location. The propensity of some arterial locations to develop stenosis might be related to factor not attributable to size *per se*, for example, anterior vs. posterior circulation. There might be some value in studying why brain arteries have differences in morphometric features that are exclusive of size *per-se* or of location. Because the anterior and the posterior circulation differ in their embryological origins, branching patterns, and flow velocities, it may be that some differences found by arterial location might be related to posterior vs. anterior circulation, although that is a secondary analysis that will also be explored in the future in the context of demographic and clinical data that we purposefully left out of this first analysis.

Studying the remodeling of brain arteries has a long history. Pioneers of brain arterial pathology have described their observations over the last centuries, with incremental sophistication in their methods (Willis et al., 1684; Morgagni, 1761; Durand-Fardel, 1854; Padget, 1944; Hassler, 1962; Fisher, 1965). However, we find many reasons to justify a renewed interest in this topic. Most of the old studies included populations with no risk factor control and therefore, likely to have exaggerated forms of arterial damage. Socioeconomic and demographic variables have changed significantly in the US and make these old findings less generalizable to the current US population, particularly to those with HIV. Finally, newer techniques to evaluate protein expression, in the form of immunohistochemistry, RNA quantitation, and proteonomics, can generate mechanistic proposals that lead to newer, target interventions to modify pathological arterial remodeling. We and others have used these newer imaging techniques to study arterial lumen- and/or wall-based remodeling in contemporary samples. However, purely lumen-based studies can underestimate the degree of arterial disease since the lumen is not usually affected until later in the remodeling process (Glagov et al., 1987). For the wall imaging studies, the imaging resolution is usually not less than 200 microns per pixel, which is not enough to decipher precisely the thickness of each arterial layer (Gutierrez et al., 2011a,b; Skarpathiotakis et al., 2013). Based on these limitations, we believe that a pathology study with a reliable arterial characterization as we have attempted is of high importance for the better understanding and treatment of cerebrovascular diseases.

Because HIV-associated brain arterial remodeling is a major focus of interest, the demographic structure of the sample reflects an attempt to match 1:1 in age and sex the cases with HIV. Comparing age- and sex-matched individuals with and without HIV in a large sample should help elucidate whether HIV infection is associated with a unique arterial pathophysiology or if the associated sociodemographic and clinical variables explain the arterial changes observed in arteries from those with HIV (Chetty et al., 2000; Connor et al., 2000; Gutierrez et al., 2012a,b). The increased life expectancy of individuals infected with HIV in the post-ARV era has led to a shift in the morbidity and mortality profile compared to those in the pre-ARV era (Hooshyar et al., 2007). Vascular disease and dementia are major contributors to morbidity and mortality in the aging HIV population, but it remains unknown to what extent HIV infections *per-se* are responsible for the increased rates of dementia and stroke as opposed to the contribution of vascular risk, comorbidities, and aging itself (Hassler, 1962; Ovbiagele and Nath, 2011; Gutierrez et al., 2013a,b). To test the hypothesis that HIV infection has an independent effect on arterial remodeling, we included individuals with HIV with age ranging from 30 to 84 years old. Forty percent are older than 40 years and 10% are older than 60 years. To explore the hypothesized contribution of HIV to pathological aging, we also included in the sample individuals with AD that would allow comparisons of arterial characteristics observed in these individuals to those with HIV.

The detailed characterization of the arteries in this study, the relatively large sample, and the variety of demographic and vascular risk factors included in the BARS are major strengths. However, autopsy-based studies are not usually considered a good source of generalizable results but rather provide accurate and more precise data from which to generate hypotheses about pathophysiology that can later be tested at a population level (Saracci, 1993; Haneuse et al., 2009). Selection bias seen in these studies is the main problem when making inferences about the general population (Jorgensen et al., 1994; Haneuse et al., 2009). To try to have a more objective idea of this bias in this study, we compared the BARS sample to the US and NYC populations than most of the tissue donors come from (Table 5). The selection bias appeared greater for the non-HIV population than for those with HIV. This might be due to the origin of the studied participants with HIV, who were captured from communities (predominantly East Harlem, The Bronx and Brooklyn) where HIV is common (2.9% of the general population, vs. 1.4% for NYC) (Gutierrez et al., 2013a,b), in a city with one of the highest rates of HIV prevalence in the US (2011). Since 42% of the BARS HIV(−) participants come from Macedonia and are predominantly non-Hispanic whites, this might confound effects related to health care system, environment and habits when compared to that of the US population. Controlling for the origin of the brains can help identifying confounding effect; however, results from the non-HIV population in the BARS should be interpreted with caution in regards to the generalizability to the US and NYC populations.

**Table 5:** **Cardiovascular profile of the studied sample compared to the US and NY populations**.

	**HIV negative (21 or older)**			HIV positive^*^		
	**BARS (present study population)**	**NHANES (2009–2010)**	**NYC-HANES (2004)**	**BARS (present study population)**	**NHANES (2009–2010)**	**NYC-HANES^†^**
Age (years)						
Mean ± SD (SE for NNAHES and NYC HANES)	55.4 ± 17	47.3 ± 0.1	46.0 ± 0.7	46.7 ± 6.8	44.0 ± 0.6	na
Median	51	46	43	47	42	na
Range	81	59	68	30	35	na
Male sex (%)	73	50 (48–51)	46 (43–48)	75	74 (57–92)	57
Ethnicity (%)						
NH whites	74	66 (58–74)	38 (36–41)	17	22 (1–50)	10
Hispanics	14	16 (9–23)	26 (24–28)	33	23.2 (15–31)	30
Black	12	11 (9–13)	23 (20–24)	50	55 (28–82)	55
Hypertension (%)	38	30 (27–33)	26 (23–28)	59	26 (8–44)	na
Diabetes (%)	15	9.0 (8–10)	8 (7–10)	16	17 (1–43)	na
Dyslipidemia (%)	19	41 (38–53)	31 (28–33)	21	38 (1–82)	na
Smoking (%)	48.5	20 (18–22)	24 (21–26)	52	36 (2–52)	na
Cocaine use (%)	6	3 (2–4)	3 (2–4)	41	20 (5–37)	na
ARV use^**^ (%)	Not applicable			53	53 (25–80)	na

* NHANES limited testing HIV serology to age range 20–59 years old.

** For autopsy population, ARV use determined at the time of death.

† Ref: Nguyen et al. AIDS. 2008 Jan 11; 22(2):281–287, unweighted estimates.

Risk factors definition: Hypertension (htn), diabetes (dm) and dyslipidemia in NHANES and NYC-HANES were limited to self-report of a physician diagnosis of htn or use of antihypertensives, self-report of a physician diagnosis of dm or use of hypoglycemic medications including insulin, and self-report of a physician diagnosis of dyslipidemia or use of hypolipemic drugs. Smoking was defined as individuals who reported smoking more than 100 cigarettes in their life time and who currently smoke either some or all days. Cocaine use was defined as present if it occurred over the year prior to the interview. HIV status in NHANES was determined by a positive ELISA HIV test in blood. The HIV results from NYC-HANES are not publicly available. For the NHANES and NYC-HANES prevalence of vascular risks factors, the estimates were weighted to account for oversampling and non-response. We used survey procedures to obtain the means and their standard errors. The analysis was carried out with SAS software, version 9.3 (SAS Institute Inc., Cary, NC).

Abbreviations: ARV, antiretroviral therapy; na, not applicable; NYC, New York City; NHANES, National Health and Nutrition Examination Survey.

Additional sources of error and bias can be inherent to the classification of vascular risk factors, dementia, and stroke. For risk factors, self-report can lead to underdiagnoses which might create either a bias or error that deviate the results toward the null hypothesis. For example, if we had used continuous measurement cutoffs to attribute vascular risk factors in the NHANES population, the rates would be higher for HTN by 6%, for DM by 2%, and for dyslipidemia by 13%. As for the BARS population, the group with HIV is part of the MHBB prospective cohort and thus, the underdiagnoses might be less than in the general population, as there is extensive and repetitive patient interview and verification through chart documentation. For the other three sources of brains, the chart review might have also decreased the rates of underdiagnoses, but it would be difficult to accurately assess this bias.

In summary, the BARS represents a large, well-characterized collection of large and penetrating brain arteries of individuals with HIV and HIV negative controls. Because the assumptions about arterial morphometric characteristics are strongly correlated with arterial size and location, controlling for these factors should provide more accurate estimates. The aims of the BARS are to better understand the factors leading to brain arterial remodeling phenotypes as a way to further advance the knowledge of brain arterial remodeling biology and discover potential new avenues to cope with the rising incident of vascular events, particularly in the aging HIV population.

## Author contributions

Jose Gutierrez: Design and conceptualization of the study, data acquisition, analysis and interpretation of the data, drafting the manuscript. Gorazd Rosoklija: Data acquisition, revising the manuscript for intellectual content. Jacinta Murray: Data acquisition. Christina Chon: Data acquisition. Mitchell S. V. Elkind: Interpretation of the data, revising the manuscript for intellectual content. James Goldman: Data acquisition, interpretation of the data, revising the manuscript for intellectual content. Lawrence Honig: Data acquisition, interpretation of the data, revising the manuscript for intellectual content. Andrew J. Dwork: Data acquisition, interpretation of the data, revising the manuscript for intellectual content. Susan Morgello: Conceptualization of the study, data acquisition, interpretation of the data, revising the manuscript for intellectual content. Randolph S. Marshall: Conceptualization of the study, data acquisition, interpretation of the data, revising the manuscript for intellectual content.

### Conflict of interest statement

NIH/NIA P50AG08702 (PI Scott Small), Title: Alzheimer's Disease Research Center at Columbia University. NIH/NIMH R25MH080663 (PI Susan Morgello), Title: The Mount Sinai Institute for NeuroAIDS Disparities. NIH/NIMH U24MH100931 (PI Susan Morgello), Title: The Manhattan HIV Brain Bank. AHA 13CRP14800040 (PI Jose Gutierrez), Title: Contribution of hiv infection to intracranial vascular. Remodeling: a case control study. The other authors declare that the research was conducted in the absence of any commercial or financial relationships that could be construed as a potential conflict of interest.
