# Corrigendum: A quantitative perspective to the study of brain arterial remodeling of donors with and without HIV in the Brain Arterial Remodeling Study (BARS)

**DOI:** 10.3389/fphys.2014.00182

**Published:** 2014-05-08

**Authors:** Jose Gutierrez

**Affiliations:** Department of Neurology, Columbia University New York, NY, USA

**Keywords:** HIV, corrigendum, arteries, brain, vessel

Individuals with HIV in this study were 140 (and not 138 as previously reported), 135 cases with HIV were obtained from the MHBB (ant not 133 as reported). The percentage of men without HIV in the study was 63 (and not 73% as reported).

## Conflict of interest statement

The author declares that the research was conducted in the absence of any commercial or financial relationships that could be construed as a potential conflict of interest.

