# Supplementary material for: A quantitative perspective to the study of brain arterial remodeling of donors with and without HIV in the Brain Arterial Remodeling Study (BARS)
Source: Front Physiol. 2014 Feb 19;5:56. doi: 10.3389/fphys.2014.00056 (PMC3928551; doi:10.3389/fphys.2014.00056)
Supplement: Supplementary file 1 [file Presentation1.PDF]

Steps for arterial segmentation: Large arteries

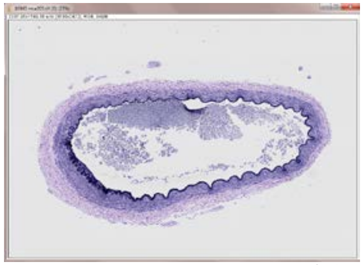

Step 1: Digital photograph of the artery (6 µm paraffin section stained with EVG).

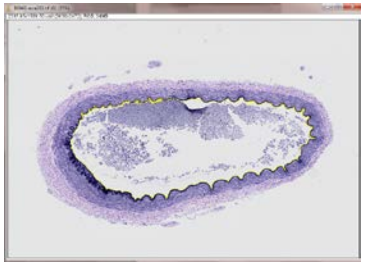

Step 2: Clear the lumen area of debris.

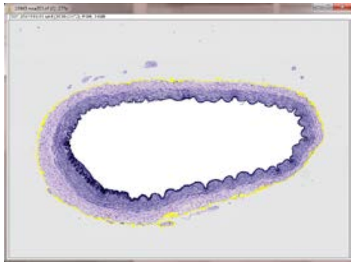

Step 3: Clear the external arterial area of debris.

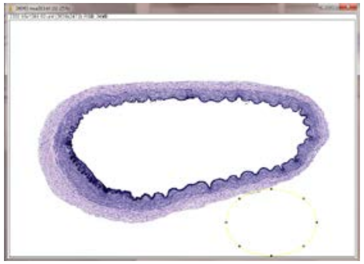

Step 4: Smoothing out the external perimeter **<u>decreases</u>** total area by **<u>5.8 ±3.7 %.</u>**

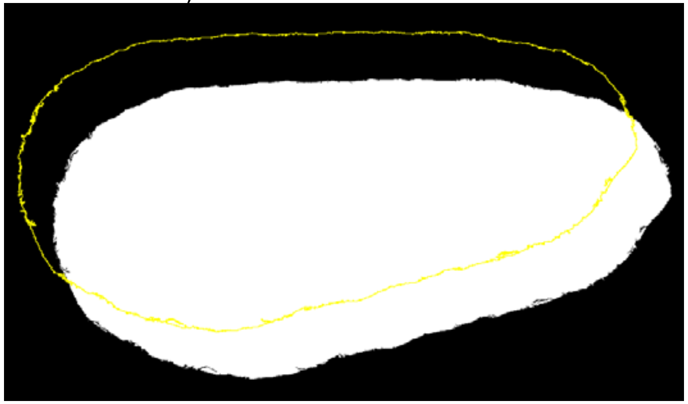

Step 5: Create a mask of the total artery area. Note rough perimeter (in yellow). Measuring the yellow line as perimeter would artificially increase the length.

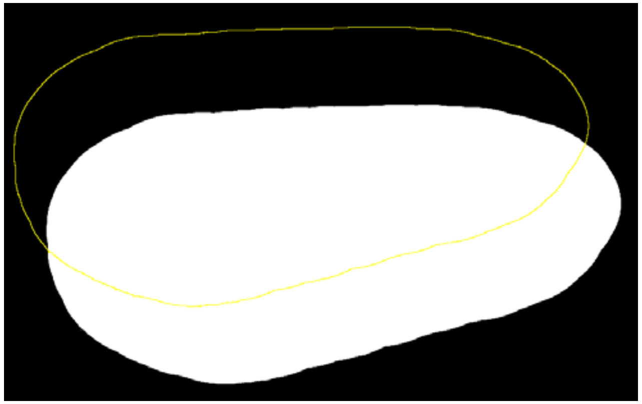

Step 6: Further smooth out the edges to obtain perimeter and total area. Smoothing out edges leads to an **<u>increase of 4.8 ± 1.4 %</u>** in total artery area.

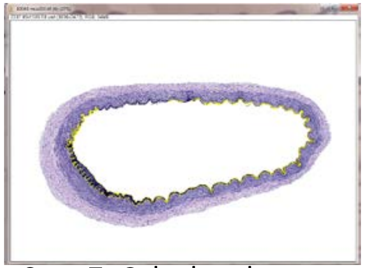

Step 7: Calculate lumen area.

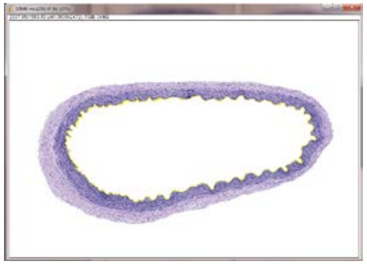

Step 8: Segment out the intima by color thresholding. Calculate the internal elastic lamina area.

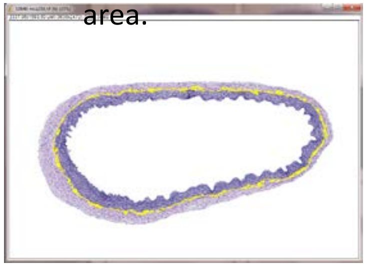

Step 9: Segment out the media by color thresholding.

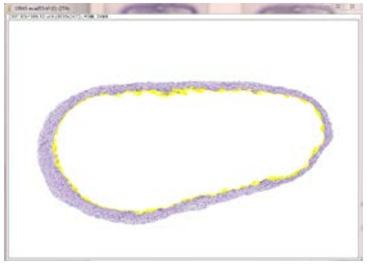

Step 10: Calculate media area.

Steps for arterial segmentation: Penetrating arteries

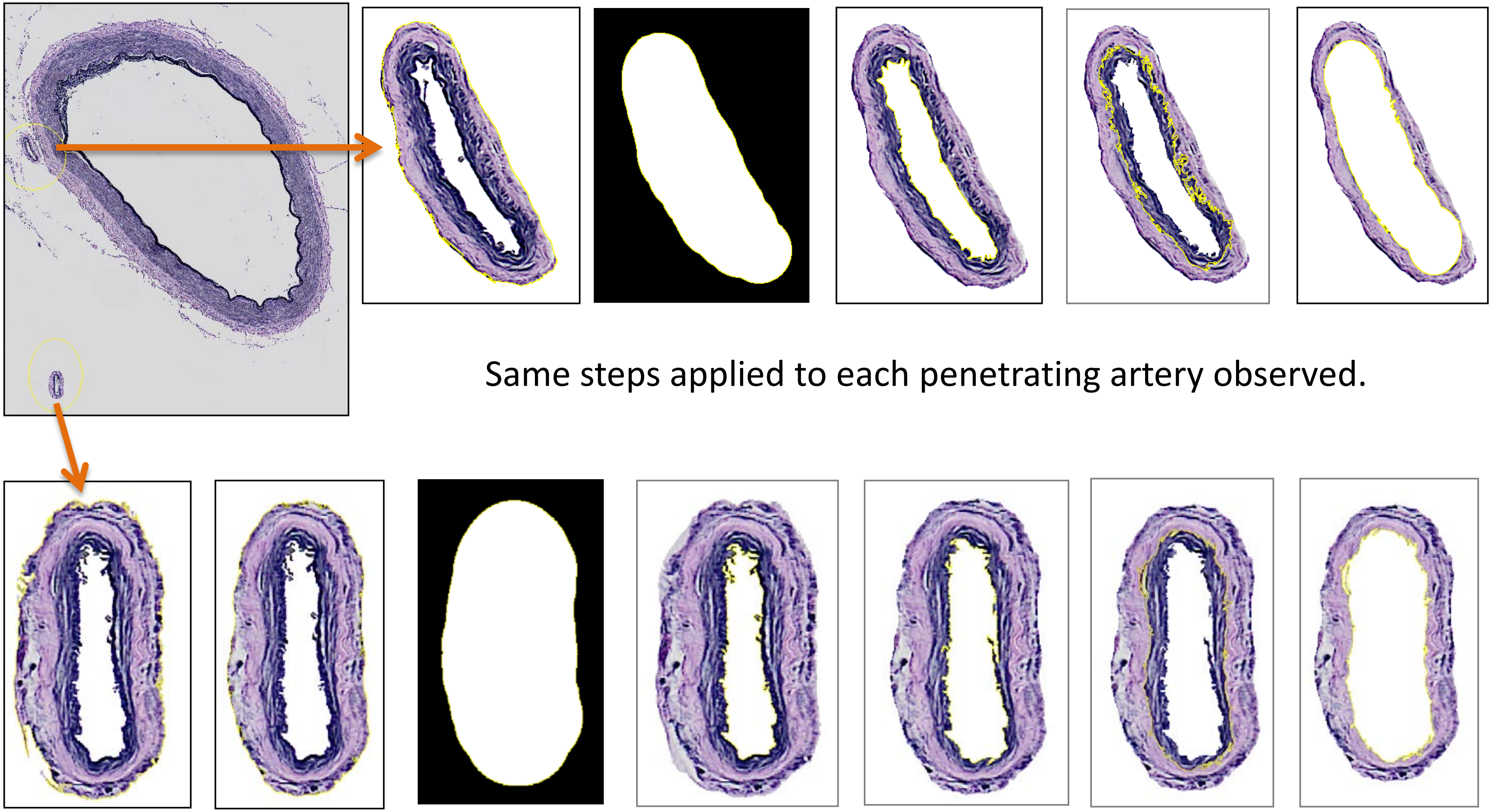

Same steps applied to each penetrating artery observed.

Step 1:
Create separate digital photograph for penetrating artery.

Step 2:
Smooth out edges **decreases** total arterial size by **9.9 ±3.3%**

Step 3:
Create mask of total artery area, smooth out edges. The total area is **increased by 5.4 ± 1.8 %** by smoothing edges of the mask.

Step 4:
Calculate lumen area.

Step 5:
Segment out intima and calculate internal elastic lamina area by color thresholding.

Step 6:
Segment out media by color thresholding.

Step 7:
Calculate media area.

Calculations of arterial characteristics:

Correcting for shrinkage artifact:

- perim_sk=Perim*1.16.
- adv_area_sk=adv_area*1.25.
- lumen_area_sk=lumen_area*1.25.
- iel_area_sk=iel_area*1.25.
- media_area_sk=media_area*1.25.

Obtaining individual layer areas:

- media=media_area_sk - iel_area_sk.
- adventitia=adv_area_sk-media_area_sk.
- intima=iel_area_sk-lumen_area_sk.
- wall_area=media+adventitia+intima.

Deriving perimeter and potential lumen areas (to correct for lumen collapse in folded arteries):

- interadv_diam=perim_sk/3.1416.
- adv_radius=interadv_diam/2.
- r_sq=adv_radius*adv_radius.
- der_tot_area=3.1416*r_sq.
- der_lumen_area=der_tot_area-wall_area.
- der_lumen_diam=SQRT((4*der_lumen_area)/3.1416).

Abbreviations: sk, shrinkage corrected; iel, internal elastic lamina; der, derived; diam, diameter; prop, proportion.

Obtaining proportion of each layer and wall

- media_prop=(media / wall_area)*100.
- adventitia_prop=(adventitia / wall_area)*100.
- iel_prop=(iel_area_sk/der_tot_area)*100.
- wall_prop=(wall_area/der_tot_area)*100.

Obtaining wall-to-lumen ratio:

- wlr=der_lumen_diam/wall_thickness.

Obtaining degrees of lumen stenosis:

- Media+adventitia_areas=adv_area_sk- iel_area_sk.
- der_iel_area=der_tot_area-med_adv_area.
- intima_lesion_area=der_iel_area-der_lumen_area.
- stenosis=(intima_lesion_area/der_iel_area)*100.

Obtaining thickness of each arterial layer:

- wall_thickness=(interadv_diam-der_lumen_diam)/2.
- adv_thickness=(wall_thickness*(adventitia_prop*0.01)).
- media_thickness=(wall_thickness*(media_prop*0.01)).
- intima_thickness=(wall_thickness*(intima_prop*0.01)).
